# Measuring cognitively demanding activities in pediatric out-of-hospital cardiac arrest

**DOI:** 10.1186/s41077-023-00253-4

**Published:** 2023-05-19

**Authors:** Nathan Bahr, Jonathan Ivankovic, Garth Meckler, Matthew Hansen, Carl Eriksson, Jeanne-Marie Guise

**Affiliations:** 1 Department of Obstetrics and Gynecology, Oregon Health and Science University, 3181 SW Sam Jackson Park Rd, L-466, Portland, OR 97239 USA; 2 Department of Pediatric Emergency Medicine, University of British Columbia, 24-1160 Nicola Street, Vancouver, BC V6G 2E5 Canada; 3 Department of Pediatrics, University of British Columbia, Vancouver, V6G 2E5 Canada; 4 Department of Emergency Medicine, Oregon Health and Science University, 3181 SW Sam Jackson Park Rd, HRC 11D01, Portland, OR 97239 USA; 5 Department of Pediatrics, Oregon Health and Science University, 3181 SW Sam Jackson Park Rd, CDRC 1231, Portland, OR 97239 USA; 6 Department of Obstetrics, Gynecology, and Reproductive Biology, Beth Israel Deaconess Medical Center and Harvard Medical School, East Campus, Kirstein 3Rd Floor, OBGYN, 330 Brookline Ave, Boston, MA 02215 USA

**Keywords:** Simulation, Pediatric out-of-hospital cardiac arrest, Emergency medical services, Cognitive load, Functional near-infrared spectroscopy

## Abstract

**Background:**

This methodological intersection article demonstrates a method to measure cognitive load in clinical simulations. Researchers have hypothesized that high levels of cognitive load reduce performance and increase errors. This phenomenon has been studied primarily by experimental designs that measure responses to predetermined stimuli and self-reports that reduce the experience to a summative value. Our goal was to develop a method to identify clinical activities with high cognitive burden using physiologic measures.

**Methods:**

Teams of emergency medical responders were recruited from local fire departments to participate in a scenario with a shockable pediatric out-of-hospital cardiac arrest (POHCA) patient. The scenario was standardized with the patient being resuscitated after receiving high-quality CPR and 3 defibrillations. Each team had a person in charge (PIC) who wore a functional near-infrared spectroscopy (fNIRS) device that recorded changes in oxygenated and deoxygenated hemoglobin concentration in their prefrontal cortex (PFC), which was interpreted as cognitive activity.

We developed a data processing pipeline to remove nonneural noise (e.g., motion artifacts, heart rate, respiration, and blood pressure) and detect statistically significant changes in cognitive activity. Two researchers independently watched videos and coded clinical tasks corresponding to detected events. Disagreements were resolved through consensus, and results were validated by clinicians.

**Results:**

We conducted 18 simulations with 122 participants. Participants arrived in teams of 4 to 7 members, including one PIC. We recorded the PIC’s fNIRS signals and identified 173 events associated with increased cognitive activity. [Defibrillation] (*N* = 34); [medication] dosing (*N* = 33); and [rhythm checks] (*N* = 28) coincided most frequently with detected elevations in cognitive activity. [Defibrillations] had affinity with the right PFC, while [medication] dosing and [rhythm checks] had affinity with the left PFC.

**Conclusions:**

FNIRS is a promising tool for physiologically measuring cognitive load. We describe a novel approach to scan the signal for statistically significant events with no a priori assumptions of when they occur. The events corresponded to key resuscitation tasks and appeared to be specific to the type of task based on activated regions in the PFC. Identifying and understanding the clinical tasks that require high cognitive load can suggest targets for interventions to decrease cognitive load and errors in care.

## Background

Cognitive errors have been implicated in causing adverse safety events that harm patients [1]. Researchers have used different methods to study cognition, with the goal of improving safety [2]. The NASA Task Load Index [3] (NASA-TLX) is a prevalent method in which subjects rate their perceived load when performing tasks. The results are easy to interpret and summarize the overall experience. Physiological measures, like heart rate, pupillary dilation, and skin conductivity, can also measure cognitive load as the body’s response to a task [4]. These methods are indirect and require more effort to interpret.

Functional near-infrared spectroscopy (fNIRS) has emerged as a physiological method to directly measure cognitive activity. An fNIRS device is worn as headgear with embedded light sources and detectors. The light sources project near-infrared light into the cranial tissues, and the detectors measure changes in oxygen concentration based on the amount of light absorbed. Higher levels of oxygen correspond to higher levels of cognitive activity. This method has distinct advantages in that it can directly and continuously measure cognitive activity in naturalistic settings. Pinti and colleagues provide an overview of the many applications of fNIRS [5]. fNIRS has historically been studied in controlled experimental designs to measure cognitive response to predetermined stimuli [6]. In these designs, subjects undergo exposure to a predetermined stimulus that is expected to evoke a hemodynamic response such as a pedestrian appearing in front of a driver. Pinti, Merla, and Aichelburg recently proposed a data-driven method to scan for functional events without prior knowledge of the stimuli [7]. This approach iteratively fits segments of fNIRS signals to the canonical hemodynamic response function (HRF) [8, 9]. Statistically significant matches are labeled as responses to unknown stimuli. This method could be useful in detecting events that increase cognitive load in dynamic clinical simulations.

The objective of this study was to explore the acquisition, cleaning, and interpretation of fNIRS data in simulated clinical scenarios to identify clinical events with high cognitive load. Identifying events associated with high cognitive load could improve understanding of threats to safety in critical situations, such as pediatric out-of-hospital cardiac arrests (POHCA).

## Methods

We conducted a cross-sectional observational study to characterize the cognitive activity of emergency medical services (EMS) providers during a simulation-based activity. Teams of paramedics and emergency medical technicians (EMTs) were recruited from local fire departments to participate in a simulated POHCA scenario. EMS teams were recruited in “engine companies” or fixed teams who typically work together based on the availability on the day of the simulation. Voluntary informed consent for participation, media release, and confidentially were obtained from participants. Each team had a person in charge (PIC) who were trained paramedics and wore the fNIRS device during the scenario. Teams underwent an orientation where they were able to familiarize themselves with the patient simulator (SimJunior® by Laerdal) and the simulated monitor (iSimulate, Albany, NY, USA) that were used in the scenario. We also surveyed the paramedics for their level of experience.

The simulations were conducted at training centers close to the participants. The setup and session facilitation were standardized. Each scenario started with a patient simulator lying supine on the floor, an iSimulate patient monitor nearby, an overhead GoPro, trained actor, and room dividers to minimize distractions. The actor portrayed a distraught family member performing CPR on the patient. The actor followed a script telling responders that the patient has developmental issues became unresponsive a few minutes ago. Clinical investigators stood behind screens to make observations and troubleshoot technical difficulties. The participants were instructed that no interaction would happen with the investigators unless there was a technical failure. For consistency, all scenarios were concluded at 10 min. Teams used their own equipment and responded to the simulations as they normally would during patient care.

### Scenario

Teams were dispatched to a 6-year-old boy who had passed out twice in the past week. The patient presented as not breathing, unresponsive, and started with a shockable rhythm. The teams were expected to perform high-quality CPR, defibrillation every 3 min, administer epinephrine after the second defibrillation, and prepare an antiarrhythmic medication after the third defibrillation according to pediatric advanced life support (PALS) guidelines [10]. The vital signs were programmed to be unchanged for the first two defibrillations and become normal after the third defibrillation.

### FNIRS device and configuration

Optical measurements were captured using a continuous wave functional near-infrared spectrometer (OctaMon, Artinis Medical Systems, The Netherlands). OctaMon is a wireless headband with eight optodes, or bundles of optical fiber, light transmitters, and receivers. The transmitters project near-infrared light through cranial tissues at wavelengths of 759 nm and 841 nm. The receivers measure how much light is absorbed at a frequency of 50 Hz. The optical density, or change in absorbed light, was converted to changes in deoxygenated (HbR) and oxygenated (HbO) hemoglobin concentration by the modified Beer-Lambert law.

**Fig. 1:**
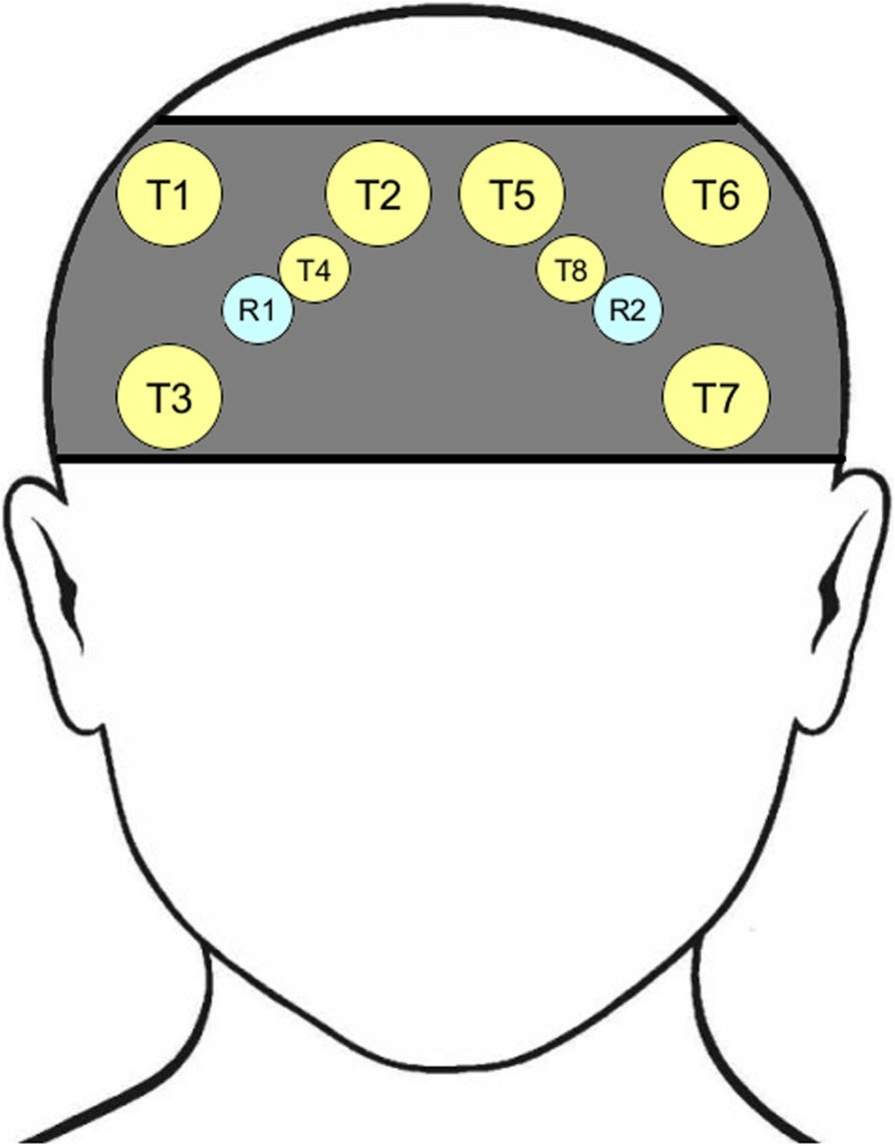
Optode layout of 3 transmitters × 2 receivers for long channels and 1 transmitter × 2 receivers for short channels

Figure 1 illustrates that the optodes were placed in a configuration of 3 × 2 long and 1 × 2 short channels over the prefrontal cortex (PFC), where channels correspond to a transmitter and receiver pair. The distance between transmitters and receivers was 35 mm in long channels and 10 mm in short channels. The long channels recorded neural and nonneural signals, e.g., heart rate, respiration, and blood pressure. The short channels recorded nonneural signals such that they could be filtered from the long channels [11].

### Data processing pipeline

**Fig. 2:**
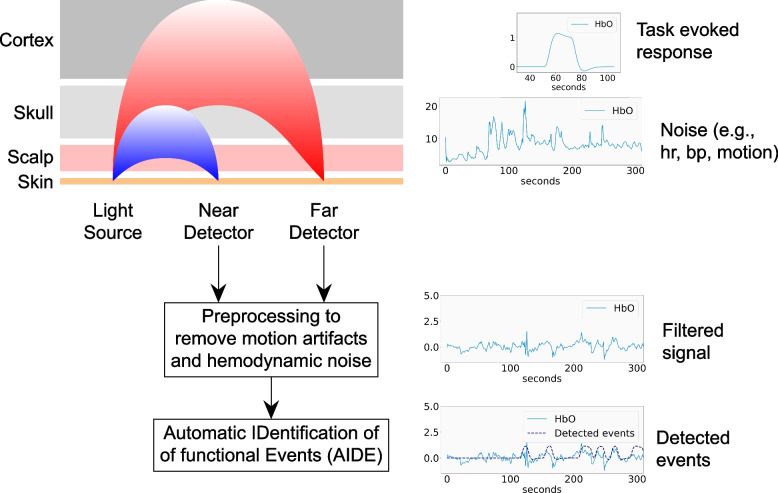
The data processing pipeline

The objective of an fNIRS analysis is to measure task-evoked hemodynamic responses. We established a data processing pipeline, summarized in Fig. 2, to determine signal quality, filter nonneural signal, and identify cognitive events. The data processing pipeline was implemented in Python 3.8 and used the Pandas 1.4.2, Numpy 1.21.5, Scipy 1.7.3, Scikit-learn 1.0.2, and Nilearn 0.7.1 libraries for numerical processing [12–16]. After processing, two researchers (N. B. and J. I.) mapped functional events to videos and coded clinical activities.

### Signal quality

We used the signal quality index (SQI) to rate our data [17]. The SQI determined if light measured by the device fell within acceptable ranges and rated quality based on the clarity of the heartbeat in the signal. The SQI was applied in 10-s windows to identify low-quality segments, channels, and samples. We used a wavelet filter [18] to correct for motion artifacts in the segments and excluded low-quality channels and samples from subsequent analysis.

### Filtering nonneural signals

After evaluating signal quality, we used recursive least-squares (RLS) adaptive filter [19] to remove nonneural components from the long channels.

1 $$e\left(k\right)=d\left(k\right)-{X}^{T}\left(k\right)W\left(k\right)$$

In equation *e(k)* = *d(k) – X*^*T*^*(k)W(k)* (1), the filtered signal *e(k)* is calculated as the difference between the signal from the long channel *d(k)* and the weighted short channel *X*^*T*^*(k)W(k)*, where *k* is an index at a given time point and *X(k)* is an nth-order vector *X(k)* = *[x(k) x(k-1) … x(k-N)]*. Weights were applied because light travels a different distance in short channels than long channels, and this affects its intensity. The weights were updated based on a least-squares error criterion and included a forget factor, set to 0.9999, which limited influence of information from the distant past. The order allowed the filter to adapt to nonstationary changes and was set to 16.

### Identification of cognitive events

**Fig. 3:**
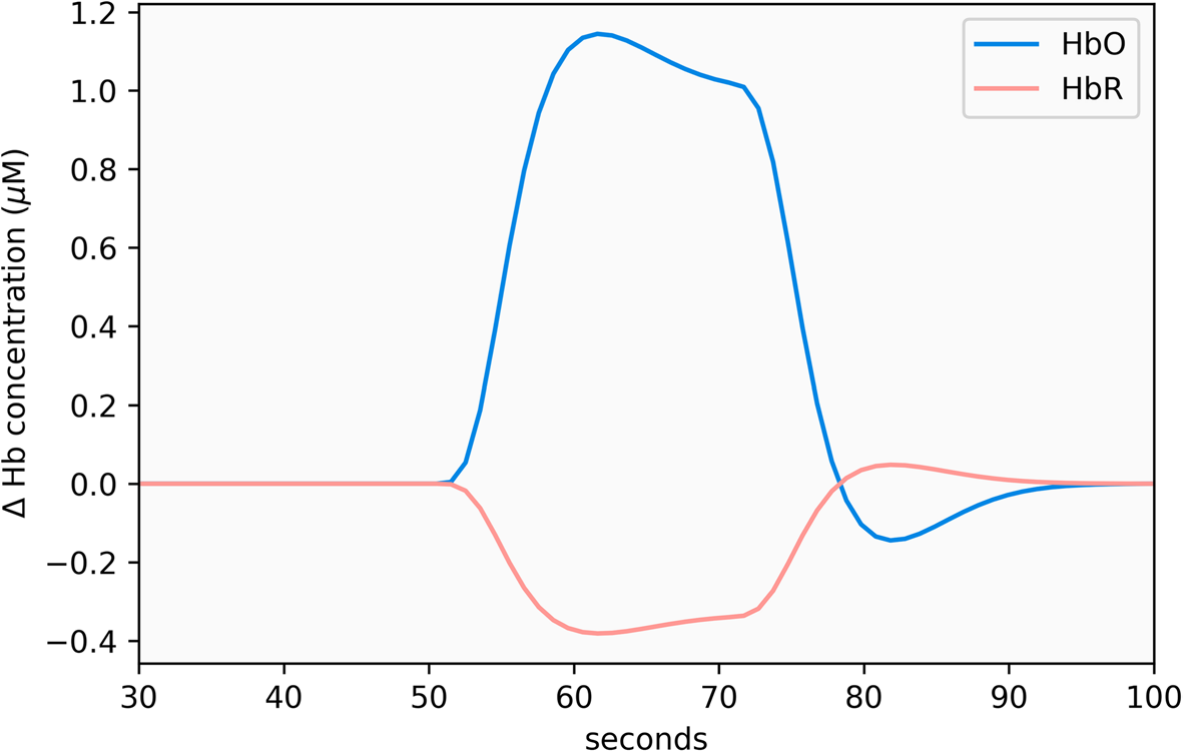
Canonical hemodynamic response function

After removing the noise, we applied the Automatic IDentification of functional Events (AIDE) algorithm [7] as an exploratory method to identify clinical events associated with increased cognitive activity. The algorithm tries to fit the HRF [8, 9] for different start times and durations. The HRF, shown in Fig. 3, models cognitive activity as an increase in HbO and decrease in HbR, followed by a reversion back to baseline. It is a mixture of gamma functions with parameters that specify peak delay *τ*_p_, undershoot delay *τ*_d_, and amplitude ratio *A* between peaks. The parameters for HbO were set to 6, 10, and 6, respectively. HbR was set as the inverse of the HbO function with − 1/3 magnitude.

2 $$h\left({\tau }_{p},t\right)=\frac{{t}^{{\tau }_{p}}{e}^{-t}}{\left({\tau }_{p}\right)!}- \frac{{t}^{{\tau }_{p}+{\tau }_{d}}{e}^{-t}}{A\left({\tau }_{p}+{\tau }_{d}\right)!}$$

There is an ongoing debate about analyzing the signals separately or combined [20]. HbO has greater signal-to-noise ratio but is more likely to be contaminated with physiological noise. A significant change in both signals potentially provides a more reliable but less sensitive indication of a cognitive event [21]. We applied the AIDE algorithm to HbO and HbR separately and coded events that co-occurred in oxygenated and deoxygenated channels.

We used generalized linear modeling (tools) from Nilearn [22] to compare the HRF to the signal for different times and durations between 10 and 30 s in length. For each time point, the duration with the best *t*-score and *p*-value was retained. We used the peak finding algorithm from SciPy [16] to identify locally maximum *t*-scores that indicated the start of functional events. Events that contained other events were excluded to favor granularity.

### Coding of clinical activities

**Fig. 4:**
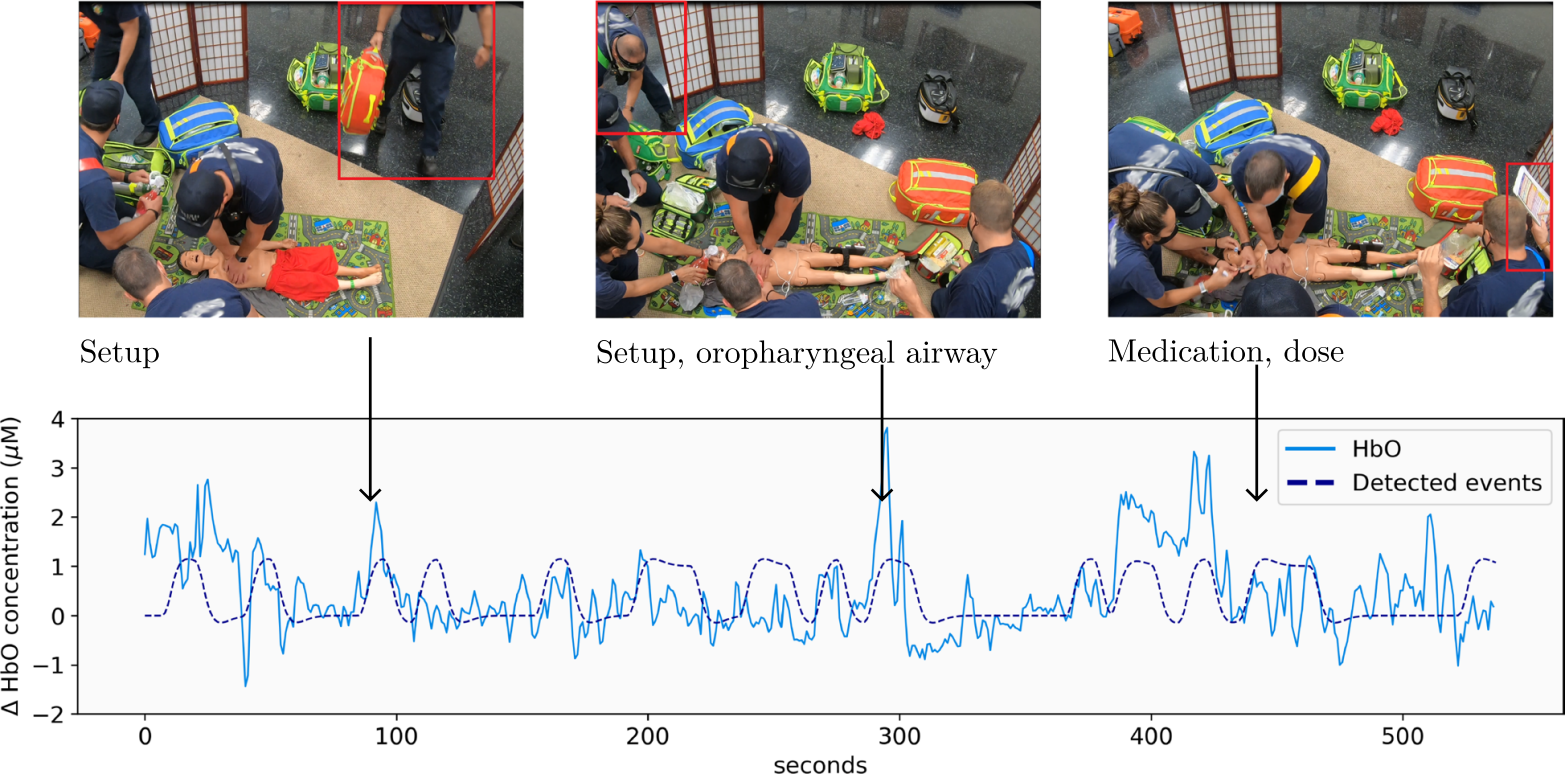
Coding analysis, add legend describing B and A type figure

The functional events were mapped to times in the video recordings, as illustrated in Fig. 4. Two researchers (N. B. and J. I.) coded clinical tasks that the PIC performed when functional events were detected in 2 or more oxygenated and deoxygenated channels. The clinical tasks included airway procedures, assessment, circulation, medication dosing and administration, recalling and following protocol, rhythm checks, setup equipment, defibrillating the patient, simulation problems, and unknown events. The criteria to code events with 2 or more active channels were established to minimize false positives.

## Results

We recorded fNIRS signals from 18 simulations at 3 different facilities. There were a total of 122 participants: 18 PICs, 58 paramedic team members, and 46 EMT team members. The PICs led teams with a mean (SD) of 6 (1) members. Table 1 describes the level of experience of the PICs and paramedic team members. PICs and team members reported having similar levels of experience and number of pediatric cardiac arrests treated in the prior year.

**Table 1:** PIC characteristics. Data are presented as mean (SD)

**PIC characteristics, *n* = 18**	
Years of experience	7.9 (6.7)
Pediatric cardiac arrests treated in the past year	1.3 (0.5)
Simulated pediatric cardiac arrests treated in the past year	1.8 (0.6)
**Paramedic team member characteristics, *n* = 58**	
Years of experience	11.6 (6.6)
Pediatric cardiac arrests treated in the past year	1.1 (0.3)
Simulated pediatric cardiac arrests treated in the past year	1.9 (0.8)

Table 2 describes the events that were detected and coded. Defibrillation, medication, and rhythm assessment were the most frequent events and considered critical tasks for the POHCA scenario. These events were not evenly distributed across all PICs and could depend on the area of care they focused on or delegated. For example, PICs had more medication events when calculating the dose themselves vs delegating that task to another teammate.

**Table 2:** Examples and frequency of detected cognitive events

Events	Examples	Frequency of detected events
Airway	Clear airway, look up equipment size for intubation	9
Assess	Collect vital signs, situational information, and history to understand the patient’s underlying problems	21
Circulation	Supervise chest compressions and swap team members to maintain quality	1
Medication	Look up, calculate dose, and administer or monitor administration of drugs	34
Protocol	Review or explain steps to provide optimal care	20
Rhythm	Pause activity to assess patient vital signs and determine next course of action	29
Setup	Unpacking, connecting, and attaching tools for resuscitation	25
Defibrillation	Recognize patient’s pulse and apply electrical shock to reset patient’s heart rhythm	34
Grand total		173

Table 3 shows the frequency of events by channel. The rhythm and medication events appear to have a strong affinity for the left PFC, whereas setup and defibrillation have an affinity for the right PFC. Other events did not exhibit a consistent response in a given hemisphere.

**Table 3:** Frequency of cognitive events by channel and hemisphere

Event	Right prefrontal cortex						Left prefrontal cortex					
	**HbO 1**	**HbR 1**	**HbO 2**	**HbR 2**	**HbO 3**	**HbR 3**	**HbO 5**	**HbR 5**	**HbO 6**	**HbR 6**	**HbO 7**	**HbR 7**
Airway	3	4	3	2	3	4	2	1	2	2	4	4
Assess	6	5	5	6	7	7	8	7	5	6	5	4
Circulation	0	0	1	1	1	0	0	0	0	0	0	0
Medication	5	3	8	4	9	7	13	16	16	10	15	16
Protocol	5	3	4	7	6	5	3	7	9	5	7	8
Rhythm	10	9	4	3	7	6	6	8	13	11	9	9
Setup	7	8	10	9	10	12	5	4	4	5	9	8
Defibrillation	13	12	13	15	10	8	12	8	9	6	9	10

## Discussion

We used a data-driven method to detect cognitive events that matched critical steps in POHCA resuscitation during dynamic clinical simulations. POHCAs are rare, high-mortality events, [23] and children pose known challenges to care due to their size and physiology [24]. The most frequently detected interventions, [defibrillation], [medication] dosing, and [rhythm] checks, corresponded to times that demanded the PIC’s attention to optimize quality of care. These events also appeared in localized regions of the brain, suggesting some functional dependency.

Our approach differs from previous fNIRS studies, which use a hypothesis-driven methodology to test if specific events exceed a predetermined threshold. Li et al. [25] mixed simulated driving with *n*-back tasks to validate that fNIRS is sensitive to workload changes in rail transit drivers. Taylor et al. [26] compared healthcare employees’ performance of tasks under varying conditions, e.g., performing a handoff in-person or over video. They concluded that fNIRS was a useful tool in measuring cognitive load, and that load increased with task complexity. This work highlights the potential for fNIRS to enhance future simulation research through objective determination of which aspects of care are associated with higher cognitive load and may be high-yield targets for education or systems improvement.

For data cleaning, different methods have been used to remove nonneural noise from fNIRS signals. Band-pass filters are the most common technique [27] but are only partially effective because the frequencies of some physiological signal overlap with the hemodynamic response [28]. We used an adaptive filter [19] with short-separation channels. Short-distance channels are considered effective for isolating and removing physiological noise [6, 29]. The adaptive aspect of the filter allowed it to compensate for nonstationary nature of the noise, in which light could travel different distances based on changes in the tissue [30]. We also applied a motion-correction wavelet filter [18]. In our initial attempt to code events, we observed that many corresponded to pronounced physical movements, such as kneeling, standing, and lifting equipment. The wavelet filter was effective to exclude these artifacts from analysis.

To detect events, we made adjustments to the AIDE algorithm. First, the length of an event was limited to 10–30 s. The original algorithm allowed events to span 1–300 s. This led to the detection of many small events or a few long events that potentially overlapped. The limit was established as a heuristic to focus on a temporal unit of analysis that fits the activity. A second adjustment was to analyze HbO and HbR separately. The original algorithm combined HbO and HbR into one activation signal [31]. Prior research has found that the signals have different sensitivities to noise, and that detecting concurrent events in both would improve specificity at the cost of sensitivity. We accepted this trade-off because in this dynamic clinical scenario, participants were physically active which increased the likelihood of noise. We coded events that were detected across multiple channels and found that they aligned with our expectations of high-cognitive load events: [defibrillation], [medication] preparation, and [rhythm] analysis corresponded to essential tasks for POHCA care. Medication events, in particular, have been well-documented in the literature as associated with high rates of error based on the need to calculate weight-based dosing [32, 33]. The affinity tasks have with left or right PFC also match mental resources that would be activated. For example, medication-related events were detected more frequently in the left PFC, which is associated with semantic and mathematical problem-solving [34, 35]. Defibrillation events involve monitoring teammates to make sure they are clear of the patient before administering an electrical charge. This event was detected more frequently in the right PFC, which is associated with visual-spatial organization and conflict detection [35, 36]. These findings demonstrate that fNIRS can detect cognitive events of clinical interest and provide insight on the modality of effort.

This work has several limitations. The clinical scenario was created and standardized by expert clinicians, but did not employ all steps described by INACSL as the gold standard for simulation scenario design [37]. Second, this was an observational study that was data driven and exploratory rather than hypothesis driven, and our observed events may contain false positives or false negatives. Finally, while we observed and report on lateralization of task-specific cognitive load, we did not account for PIC handedness which may relate to functional brain mapping [38, 39].

High cognitive load does not necessarily mean that performance will be low or errors will occur. For example, the Yerkes-Dodson curve [40] suggests that there is an optimal load, between disinterest and overburden, at which performance is maximized. This could be explored by comparing cognitive load to errors in simulation and topics discussed in debriefing. These methods and findings could be useful to EMS educators in terms of adjusting the difficulty of training to improve engagement and retention of knowledge.

## Conclusion

Detected events in fNIRS signals appeared to coincide with critical tasks in POHCA simulations and have potential for measuring cognitive load in dynamic clinical situations. We used the data-driven algorithm, AIDE, and detected critical tasks that were performed during POHCA care. We also obtained insight on the cognitive demands of these tasks, based on the activated regions of the prefrontal cortex. Future work may involve further refining data cleaning and interpretation through analysis of public data sets and validating our findings across a range of clinical scenarios with the goal of identifying targets to mitigate the impact of cognitive load on clinical practice during high-stake, low-frequency events.

## Data Availability

The datasets used and/or analyzed during the current study are available from the corresponding author on reasonable request.

## Abbreviations

AIDE: Automatic identification of functional events
EMS: Emergency medical services
EMT: Emergency medical technician
fNIRS: Functional near-infrared spectroscopy
HbO: Oxygenated hemoglobin concentration
HbR: Deoxygenated hemoglobin concentration
HRF: Hemodynamic response function
PALS: Pediatric advanced life support
PFC: Prefrontal cortex
PIC: Person in charge
POHCA: Pediatric out-of-hospital cardiac arrest
RLS: Recursive least-squares adaptive filter
SQI: Signal quality index
